# Obesogenic television food advertising to children in Malaysia: sociocultural variations

**DOI:** 10.3402/gha.v7.25169

**Published:** 2014-08-19

**Authors:** See H. Ng, Bridget Kelly, Chee H. Se, Karuthan Chinna, Mohd Jamil Sameeha, Shanthi Krishnasamy, Ismail MN, Tilakavati Karupaiah

**Affiliations:** 1 Dietetics Program, School of Healthcare Sciences, Faculty of Health Sciences, National University of Malaysia, Kuala Lumpur, Malaysia; 2 Early Start Research Institute, Faculty of Social Sciences, University of Wollongong, Wollongong, Australia; 3 Epidemiology and Biostatistics Unit, Department of Social and Preventive Medicine, Faculty of Medicine, University of Malaya, Kuala Lumpur, Malaysia; 4 Nutrition Program, School of Healthcare Sciences, Faculty of Health Sciences, National University of Malaysia, Kuala Lumpur, Malaysia; 5 Nutrition & Dietetics Department, Faculty of Health Sciences, UiTM, Puncak Alam, Malaysia

**Keywords:** content analysis, food marketing, television, sugar-sweetened drink, obesogenic environment

## Abstract

**Background:**

Food advertising on television (TV) is well known to influence children's purchasing requests and models negative food habits in Western countries. Advertising of unhealthy foods is a contributor to the obesogenic environment that is a key driver of rising rates of childhood obesity. Children in developing countries are more at risk of being targeted by such advertising, as there is a huge potential for market growth of unhealthy foods concomitant with poor regulatory infrastructure. Further, in developing countries with multi-ethnic societies, information is scarce on the nature of TV advertising targeting children.

**Objectives:**

To measure exposure and power of TV food marketing to children on popular multi-ethnic TV stations in Malaysia.

**Design:**

Ethnic-specific popular TV channels were identified using industry data. TV transmissions were recorded for each channel from November 2012 to August 2013 (16 hr/day) for randomly selected weekdays and weekend days during normal days and repeated during school holidays (*n=*88 days). Coded food/beverage advertisements were grouped into core (healthy), non-core (non-healthy), or miscellaneous (unclassified) food categories. Peak viewing time (PVT) and persuasive marketing techniques were identified.

**Results:**

Non-core foods were predominant in TV food advertising, and rates were greater during school holidays compared to normal days (3.51 *vs* 1.93 food ads/hr/channel, *p<*0.001). During normal days’ PVT, the ratio of non-core to core food advertising was higher (3.25 food ads/hr/channel), and this more than trebled during school holidays to 10.25 food ads/hr/channel. Popular channels for Indian children had the lowest rate of food advertising relative to other ethnic groups. However, sugary drinks remained a popular non-core product advertised across all broadcast periods and channels. Notably, promotional characters doubled for non-core foods during school holidays compared to normal days (1.91 *vs* 0.93 food ads/hr/channel, *p<*0.001).

**Conclusions:**

This study highlights non-core food advertising, and predominantly sugary drinks are commonly screened on Malaysian TV channels. The majority of these sugary drinks were advertised by multinational companies, and this observation warrants regulatory attention.

Food marketing is an important environmental and contextual factor influencing eating behaviours and is a worldwide public health concern (1). Within marketing, food promotion is a form of communication designed to increase the recognition, appeal and/or consumption of specific food products (2). In this communication environment, television (TV) is a major source of children's exposure to food advertisements (1, 3). TV advertising has long been recognised as an effective medium to reach out to children by food industry and is a primary promotional channel for food marketers (4, 5). Exposure and the power of marketing are two important elements to assess the impact of food marketing on children, as emphasised by the World Health Organization (WHO) (1). ‘Exposure’ is defined as the number of times a viewer is exposed to a message, whereas ‘power’ is defined as a food advertiser's technique to target young and impressionable consumers through the use of promotional characters and premium offers (1, 6). Although content analyses of TV food marketing in developed countries have provided information on the use of the elements of exposure and power in advertisements targeting children (7), such data in developing countries are still scarce.

Gorn and Goldberg (8) were the first to experimentally demonstrate that daily exposure to televised candy advertisements could influence children choosing candy over fruits. The United States National Health Examination Survey indicated that for both young and teenage children, the amount of time spent watching TV was linked to prevalence of obesity (9). Subsequently, systematic reviews have provided modest evidence showing food marketing generates positive beliefs, affects nutrition knowledge, and influences children's food preferences and food consumption patterns, as well as strong evidence that marketing enhances purchase requests to parents (4, 10– 12) . It is proposed that the impact of food promotion on children in developing countries may be greater compared to those in developed countries (13). Such children may be less familiar with advertising and less practiced in navigating commercial messages. The growing middle classes in emerging market economies such as China, India, and many Southeast Asian countries provide unparalleled growth opportunities for global multinational food companies who need to generate new growth after saturation in developed markets (14).

Indeed, it is noted that the world's food system is not a competitive marketplace of small or local producers but driven by multinational food companies (15). It has been documented that in developed countries, marketing strategies employed by multinational food companies target young people to become lifelong consumers and influence household purchases (4, 16). The excessive consumption of often energy-dense ultra-processed foods is blamed for the rising obesity epidemics and incidence of non-communicable diseases (NCDs) in Western nations (17). Of concern is that such marketing strategies, when transplanted to developing countries, would also ultimately result in these health issues if expansion of trade, foreign direct investments, and transnational food corporations proliferate in emerging economies (18). Yet, public health professionals have responded slowly to such nutritional threats in developed countries and even slower in developing countries (15). In a transitional society such as Malaysia which has witnessed economic expansion in the last three decades, overall childhood obesity prevalence in 2006 was reported to be 19.9% in Malaysia, but prevalence patterns by ethnicity were indicated to be 26.6% in Chinese, 26.1% in Indian, and 18.9% in Malay communities (19).

The obesogenic environment in Malaysia is poorly defined. Multinational food companies’ signatories to the International Food and Beverage Alliance (IFBA) have a presence in Malaysia (20). But the specific regulatory criteria promoted by IFBA appear to be permissive in the type of foods suitable for advertising to children (*authors’ opinion)*. A preliminary study in Malaysia conducted in 2006–2007 highlighted that a large proportion of TV advertising (56%) promoted foods high in fat, refined sugars, and salt (21). Concurrent to the time period of this study, Malaysian government guidelines restricted advertising and sponsorship by fast-food companies during children's TV programs (22). In this study, the majority of food advertisements broadcasted on local TV channels were snack foods, dairy products, confectionary, biscuits, and fast food. The limitation of this study was advertising data were provided by participating TV stations rather than adapting live telecast recordings as an independent approach. The method for data analysis changed with a recent study employing live recording in Singapore (5) and evaluating persuasive marketing techniques used by the food industry in Australia (6). Given the evolution of assessments over time in TV food marketing, a need is suggested to adopt new methodologies to effectively evaluate the local TV food marketing scenario.

This study aimed to measure exposure and power of TV food marketing to children in Malaysia, which is a multi-ethnic, developing country. We expect that the outcomes from this study will contribute to existing evidence on the obesogenic environment in three ways: 1) it will explore variations in advertising patterns with seasonal variation (normal days *vs* school holidays) which is recognised as an evidence gap (21, 23) and peak versus non-peak viewing time; 2) it will explore differences in marketing techniques used to target different cultural groups as highlighted by previous researchers (5, 24); and 3) it will explore the use of persuasive techniques in food advertising.

## Methods

### TV channel identification

Popular channels were identified based on ethnic-specific viewership data generated by Nielsen's Television Audience Measurement (TAM) (25). The channels deemed popular were determined through TV viewer rating. Ratings for a 1-week period (9–15 October 2011) identified the most popular channels for children aged 4–14 years based on three major ethnic groups – Malay, Chinese, and Indian. Viewership share (%) of channels was defined as the proportion of individuals’ viewership per channel compared to the total viewership for all channels for same time period (25). Based on these criteria for popular viewership, selected channels were free-to-air (FTA) and satellite TV (Pay-TV) channels that had a household penetration of more than 50%. Selected Pay-TV channels were Astro Cartoon Network (CTWK), Astro Hua Hee Dai (HHD), Astro Wah Lai Toi (WLT), Astro SUN TV (Sun TV), Astro Adithya and Astro Vellithirai (VT). FTA channels excluded were Al-Hijrah and TV1. The majority of the selected channels were not exclusive to children viewership except for the CTWK channel. However, as per TAM data, these channels were still drawing the largest child audience (data not shown). Overall 103 TV channels were assessed by Nielsen's TAM ratings to identify the ethnic-specific popular channels to be used in the data sampling.

### Data sampling

The TV sampling method has been described elsewhere (26, 27). Transmissions from live TV channels were recorded onto hard discs. In brief, TV transmissions were recorded using a personal video recorder (PVR, Kworld Analog TV Card II, Taipei, Taiwan) and software (Windows Media Centre) between 06:00 and 22:00 hours daily (16 hr/day) on randomly selected days falling between November 2012 to August 2013. For each channel, transmissions were recorded for two weekdays and two weekend days in a week during normal days and this cycle was repeated during the school holiday seasons (*n=*88 days). Normal days were defined as schooling days in Malaysia which excluded public holidays or large sporting competitions. This 10-month recording period excluded the Muslim month of *Ramadhan* and *Syawal* which otherwise has been noted to cause variation in advertising exposure (21). The Hindu festive season of *Diwali* also falls outside of the recording period. However, *Christmas* and *Chinese New Year* did fall within the stated 10-month recording period. In Malaysia, *Christmas* is only a single public holiday, and there was no change to advertising patterns observed. *Chinese New Year* has a greater impact on TV advertising patterns, particularly for the popular Chinese TV channels related to a significantly large Chinese population in Malaysia. Therefore, recording transmission data was stopped 2 weeks before Chinese New Year.

### Data coding

**Fig. 1:**
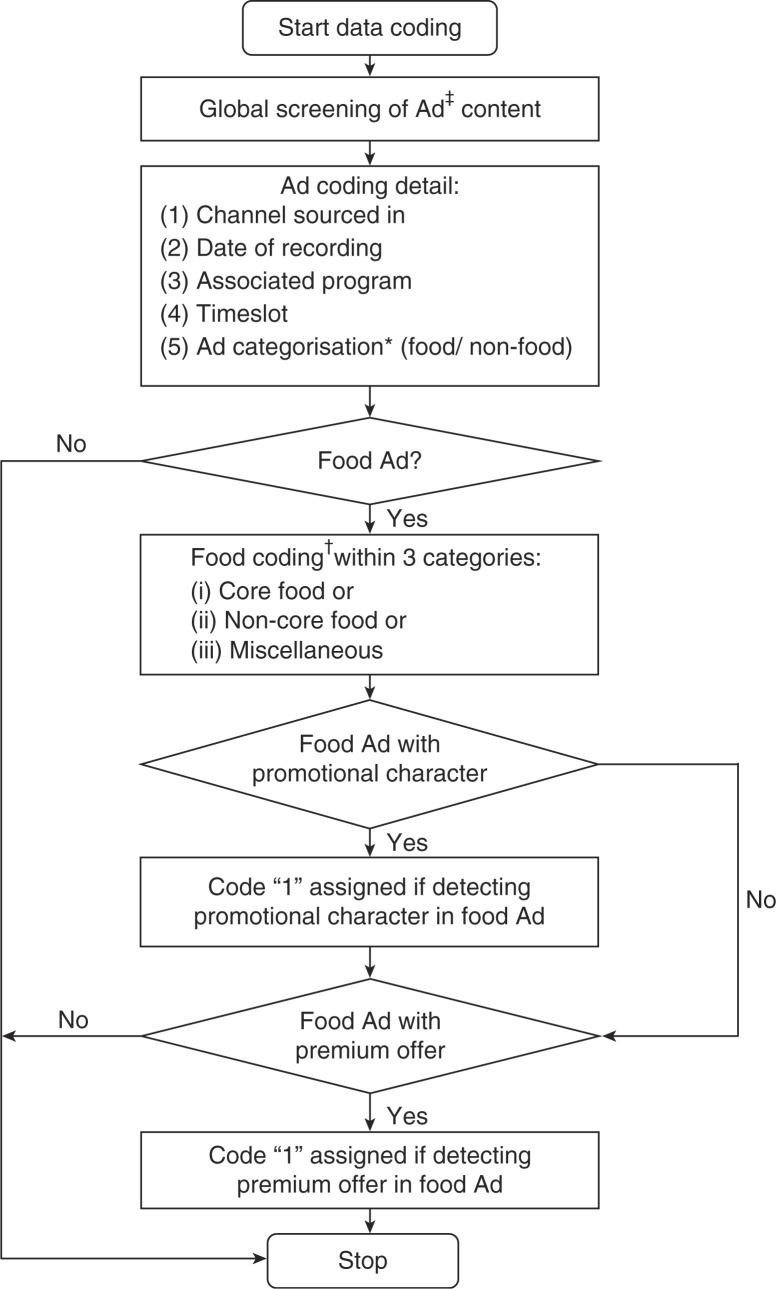
Process algorithm of data coding. Ad: advertisement. *Code of product, e.g. retail food and drink, channel promotion, education etc. ^†^Protocol with 36 food codes developed based on previous international methodology for TV food advertising (26).

Recorded TV transmissions were visually screened for advertising content to identify advertisements which were subsequently coded as per protocol outline in Fig. 1. This protocol is based on criteria described elsewhere (26). Each advertisement was coded for channel identity, date, program details, time slot at which a particular advertisement was broadcasted, and the nature of the product advertised (e.g. retail food and drink, channel promotion, education). All advertisements for retail food/beverage products, supermarkets, and restaurants were further coded into 36 food codes (Table 1) that were each assigned to one of three food categories (core, non-core, and miscellaneous foods). Core foods are nutrient dense and low in discretionary energy and can be recommended to be consumed daily, while non-core foods are high in undesirable nutrients such as high fat, refined sugars, and salt (26). Modifications were made related to food products classification relevant to the Malaysian food supply. For example, sweetened or flavoured milk would be classified as non-core food, whereas non-sweetened milk beverages would be considered as core food. If more than one food product was advertised, the first shown product or the most dominant food product was coded. All food and beverage advertisements were further evaluated for the use of persuasive techniques, including: 1) promotional characters (e.g. cartoons, celebrity endorsers) and 2) premium offers (e.g. giveaways, competitions, contests, vouchers, and rebates).

**Table 1:** Seasonal variation in 11 popular TV channels targeting children

		Rate of food advertising (food ads/hr/channel)		
		
	Overall mean (food ads/hr/channel)	Normal days^a^	School holidays	*p*
Non-core foods^b^	2.73	1.93	3.53	<0.001
Sugar-sweetened drinks	0.80	0.43	1.16	
Sweet breads/cakes/muffins/buns/biscuits, glutinous rice balls/cakes/pudding, high-fat savoury biscuits, pies, pastries	0.29	0.29	0.29	
Fast food (not only healthier options advertised)	0.28	0.26	0.29	
Savoury snack foods (added salt or fat) – chips, dried spicy peas, fruit chips, savoury crisps, extruded snacks, popcorn (exclude plain), salted or coated nuts, other fried snacks	0.20	0.13	0.27	
Chocolate and candy	0.17	0.15	0.19	
Flavoured/fried instant rice and noodle products	0.16	0.13	0.19	
Ice cream, iced confection, and desserts	0.15	0.10	0.20	
Flavoured or dairy products with added sugar and alternatives	0.13	0.07	0.19	
High-sugar and/or low-fibre breakfast cereals (>20 g sugars/100 g or <5 g dietary fibre/100 g)	0.13	0.07	0.18	
Meat and meat alternatives processed/preserved in salt	0.11	0.14	0.08	
High-fat/salt meals – frozen, packaged meals (>6 g saturated fat/serve, >900 mg sodium/serve)	0.09	0.06	0.12	
Sweet snack foods – jelly, sugar-coated dried fruits or nuts, nut/seed based bars and slices, sweet rice bars, and tinned fruit in syrup	0.09	0.05	0.13	
Fruit juice/drinks (<98% fruit)	0.08	0.03	0.13	
Other high-fat/salt products – high-fat savoury sauces (>10 g fat/100), soups (>2 g fat/100 g; all dehydrated)	0.07	0.04	0.10	
Alcohol	0.00	0.00	0.00	
Core foods^c^	0.42	0.45	0.39	0.073
Plain milks and yoghurts, cheese, and alternatives	0.13	0.16	0.10	
Breads, rice, and rice products without added fat, sugar, or salt	0.08	0.09	0.07	
Low-sugar, high-fibre breakfast cereals (<20 g sugar/100 g and >5 g fibre/100 g)	0.06	0.07	0.06	
Healthy snacks – <600 kJ/serve, <3 g saturated fat/serve, and <200 mg sodium/serve	0.06	0.05	0.06	
Oils high in mono- or polyunsaturated fats, and low-fat sauces (<10 g fat/100 g)	0.04	0.05	0.03	
Fruits and fruit products without added fats, sugars, or salt	0.02	0.02	0.03	
Meat and meat alternatives	0.01	0.01	0.02	
Water	0.01	0.00	0.02	
Vegetables and vegetable products without added fats, sugars, or salt	0.00	0.00	0.00	
Low-fat/salt meals: meals (≤6 g saturated fat/serve, ≤900 mg sodium/serve), soups (<2 g fat/100 g, exclude dehydrated), sandwiches, mixed salads	0.00	0.00	0.00	
Baby foods (exclude milk formulae)	0.00	0.00	0.00	
Miscellaneous foods/food-related^d^	0.75	0.74	0.76	0.396
Vitamin/mineral or other dietary supplements, and sugar-free chewing gum	0.28	0.27	0.30	
Recipe additions (including soup cubes, oils, dried herbs, and seasonings)	0.16	0.19	0.14	
Baby and toddler milk formulae	0.16	0.11	0.21	
Tea and coffee	0.07	0.08	0.07	
Fast-food restaurant (no foods or beverages advertised)	0.05	0.05	0.04	
Supermarkets (non-core foods advertised)	0.02	0.04	0.00	
Supermarkets (no foods or beverages advertised)	0.00	0.00	0.00	
Fast food (only healthier options advertised)	0.00	0.00	0.00	
Local restaurant	0.00	0.00	0.00	
Supermarkets (only core and healthy foods advertised)	0.00	0.00	0.00	
Ratio of non-core:core	6.54	4.32	9.11	

a Normal days: schooling days exclude national holidays, large sporting competitions, special event and public holiday in Malaysia.

b Food that is relatively high in undesirable nutrients such as high fat, refined sugars, and salt.

c Food that is recommended to be consumed daily to meet nutrient requirements.

d Food that is added to flavour meals (e.g. recipe additions); supplements; milk formula for baby and toddlers; tea and coffee (plain); fast food (with no non-core foods); or local restaurant and supermarkets.

Advertisements were coded by three researchers. To ensure consistency in data coding, an inter-coder reliability test was carried out between researchers based on 1) the number of food advertisements recorded and 2) the food code recorded, using an 1-hour random identical sample of TV data (28). The inter-coder reliability was 100% for both the number of food advertisements recorded, and food coding too.

### Data interpretation

Coded datasets were cleaned and food codes were validated by three professionals (SCH, MJS, KS) with nutrition and dietetic knowledge. Differences in professional opinion were analysed and resolved by an expert panel (BK, TK). Children's peak viewing time (PVT) was defined as the period of the day when ≥25% of the maximum children's audience were likely to be watching TV (26) as defined by Nielsen TAM data for weekday (from 19.00 to 22.00 hours) and weekend (from 15.00 to 16.00 hours and 19.00 to 22.00 hours) periods (29). Viewing time outside the defined PVT was non-peak viewing time (NPVT). Aggregation of each sample was performed to determine the count of advertisements in an hour as described by previous research (30). The average number of food advertisements per hour (rate) was calculated for each food category (core, non-core, and miscellaneous). Rates of core and non-core food advertising were assessed for normal days versus school holidays, for peak versus non-peak viewing times, and also to observe if usage of persuasive techniques in food marketing influenced these rates.

### Statistical analysis

As the rates of food advertisements over time did not fulfil normality assumptions, non-parametric analyses (Mann-Whitney U test) was used to examine seasonal differences between rates of core and non-core food advertising. Kruskal-Wallis test was applied to detect differences between ethnic channels for both normal days and school days. Post hoc analysis for pairwise comparisons between channels was carried out using Dunn Test with Bonferroni correction. A *p-*value threshold of 0.05 was used to determine statistical significance for all data analysis. The statistical analysis was conducted using IBM Statistical Package for Social Sciences, version 19.0 (IBM SPSS Statistics Inc., Chicago, IL).

**Fig. 2:**
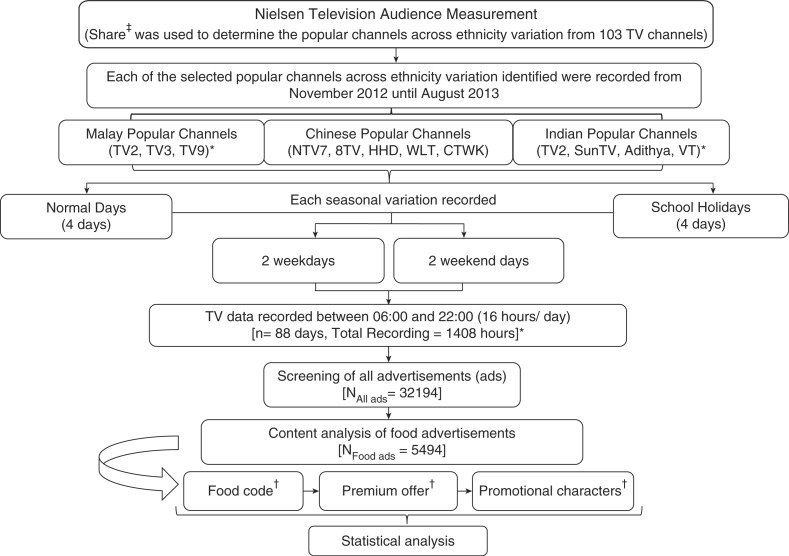
Flow chart of content analysis. For ethnic-specific popular channels, three popular channels were for Malay whilst five popular channels were Chinese and four popular channels were Indian. *However, one TV channel was common to both Malay and Indian viewership. Hence, overall analysis was carried out based on only 11 channels. ^‡^Proportion of individuals’ viewership per channel compared to the total viewership for all channels for same time period. ^†^Protocol developed based on previous international methodology for TV food advertising (26).

Figure 2 summarises the flow process of content analysis carried out for this study.

## Results

From 103 TV channels identified through Nielsen data, 11 popular channels were related to ethnicity. Malay popular channels were TV2, TV3, and TV9 whilst NTV7, 8TV, HHD, WLT, and CTWK were Chinese and Sun TV, VT, and Adithya were Indian popular channels. One channel (TV2) was common to both Malay and Indian ethnic groups. A mean rate of 3.90 food ads/hr/channel across the 11 children's popular channels in Malaysia was established. Of the 1,408 hours of TV broadcasting that were analysed, a total of 32,194 advertisements were identified of which 5,494 were for food (17.1%).

### Food advertising patterns: normal days versus school holidays

Table 1 provides distribution data for rates of food advertising as differentiated between normal days and school holidays for non-core, core, and miscellaneous food categories. The greatest frequency of food advertising rates associated with non-core foods, irrespective of normal days or school holidays. The rate of non-core food advertising was significantly higher on school holidays compared to normal days (3.53 *vs* 1.93 food ads/hr/channel; *U=*205,492; *p<*0.001) whilst the advertising rate was not significantly different (*p>*0.05) between these days for core or miscellaneous foods. Differences in the rates of non-core food advertising over these periods were attributed to sugar-sweetened drinks, for which the rate of advertising trebled during school holidays (0.43 *vs* 1.16 food ads/hr/channel, *p<*0.001). Similar patterns of exposure for sugar containing snacks like sweet breads, cakes, biscuits (both 0.29 food ads/hr/channel, *p>*0.05), fast foods (0.26 *vs* 0.29 food ads/hr/channel, *p>*0.05), chocolate and candy (0.15 *vs* 0.19 food ads/hr/channel, *p>*0.05) and savoury snack foods (0.13 *vs* 0.27 food ads/hr/channel, *p<*0.01) were detected for both normal days and school holidays. Notably, alcohol was the only non-core product found not advertised at any time, and this is probably attributed to religious restriction in a Muslim dominant country. The mean rates of advertising for core food categories (0.42 food ads/hr/channel) and miscellaneous foods (0.75 food ads/hr/channel) were lower compared to the non-core food rate (2.73 food ads/hr/channel). For every core food advertisement shown, there were nearly four non-core food advertisements shown during normal days, and this figure increased to nine during school holidays. As there was a significant difference in advertising pattern by seasonal variation, the following analyses were conducted separately for normal days and school holidays.

### TV food advertising during children's peak versus non-peak viewing times

Rates of food advertising were consistently higher during children's PVT across all food categories (Table 2). The intensity of non-core food advertising was highest during children's PVT for both normal days (2.62 *vs* 1.74 food ads/hr/channel; *U=*34,504; *p<*0.001) and school holidays (4.53 *vs* 3.26 food ads/hr/channel; *U*=33,276; *p<*0.001). The ratio of non-core:core food advertising during children's PVT was 3.25 during normal days and 10.25 during school holidays. Additionally, there were consistently greater non-core food and low-core food exposures observed in both children's PVT and NPVT during school holidays, resulting in higher non-core to core ratios during these periods (10.25 *vs* 8.74 food ads/hr/channel). These patterns were also reflected when the channels were analysed based on ethnicity. For Malay channels, the non-core foods intensified during school holidays irrespective of PVT or NPVT periods.

**Table 2:** Rate of food advertising during normal days and school holidays as defined by viewing time and ethnic nature of channels

	Rate of food advertising (food ads/hr/channel)					
	
	Normal days			School holidays		
	
	PVT	NPVT	*p*	PVT	NPVT	*p*
Overall popular channels (*n=*11)						
Non-core foods	2.62	1.74	<0.001	4.53	3.26	<0.001
Core foods	0.81	0.35	<0.001	0.44	0.37	0.114
Miscellaneous foods	1.47	0.54	<0.001	1.28	0.61	<0.001
Ratio of non-core:core	3.25	5.01		10.25	8.74	
Malay popular channels (*n=*3)^a^						
Non-core foods	3.12	2.03	0.013	7.36	5.84	0.052
Core foods	0.83	0.30	0.001	0.48	0.48	0.897
Miscellaneous foods	0.98	0.66	0.155	1.07	0.57	0.002
Ratio of non-core:core	3.74	6.78		15.45	12.17	
Chinese popular channels (*n=*5)						
Non-core foods	3.71	2.58	0.005	5.03	3.61	0.003
Core foods	1.03	0.52	<0.001	0.67	0.53	0.080
Miscellaneous foods	2.03	0.66	<0.001	1.91	0.82	<0.001
Ratio of non-core:core	3.61	4.99		7.49	6.83	
Indian popular channels (*n=*4)^a^						
Non-core foods	0.75	0.42	0.044	2.34	1.28	<0.001
Core foods	0.39	0.12	<0.001	0.05	0.04	0.527
Miscellaneous foods	0.80	0.19	0.001	0.41	0.27	0.032
Ratio of non-core:core	1.91	3.65		43.64	36.57	

PVT: peak viewing time of children; NPVT: non-peak viewing time of children.

For ethnic-specific popular channels, three popular channels were for Malay whilst five popular channels were Chinese, and four popular channels were Indian.

a However, one TV channel was common to both Malay and Indian viewership. Hence, overall analysis was carried out based on only 11 channels.

### Food advertising exposure by ethnicity

Generally, Indian channels had the lowest rate of food advertising relative to Malay and Chinese channels as indicated in Table 3. During normal days, Chinese and Malay channels broadcasted similar rates for non-core food advertising (2.83 and 2.27 food ads/hr/channel, respectively) but Indian channels had a significantly lower rate (0.49 food ads/hr/channel; *p<*0.001). In contrast, exposure to non-core food advertising on Malay channels increased almost three times to 6.17 food ads/hr/channel during school holidays and was significantly higher (*p<*0.001) compared to Chinese (3.92 food ads/hr/channel) and Indian (1.51 food ads/hr/channel) channels. Additionally, sugar-sweetened drinks remained as the most commonly advertised food product in the non-core food category across all ethnics’ popular channels (data not shown). Patterns of exposure to core and miscellaneous food advertisements during normal days and school holidays did not differ and remained relatively low for all ethnic groups’ popular channels compared to non-core food advertising rates.

**Table 3:** Food advertising exposure as per seasonal variation by TV channel ethnicity

	Rate of food advertising (food ads/hr/channel)							
	
	Normal days				School holidays			
	
	Malay channels (*n=*3)^a^	Chinese channels (*n=*5)	Indian channels (*n=*4)^a^	*p*	Malay channels (*n=*3)^a^	Chinese channels (*n=*5)	Indian channels (*n=*4)^a^	*p*
Non-core foods	2.27^a^	2.83^b^	0.49^a^^b^	<0.001	6.17^a^^b^	3.92^bc^	1.51^a^^bc^	<0.001
Core foods	0.42^a^^b^	0.63^bc^	0.18^a^^bc^	<0.001	0.48^a^	0.56^b^	0.04^a^^b^	<0.001
Miscellaneous foods	0.73^a^	0.96^b^	0.32^a^^b^	<0.001	0.68^a^	1.06^b^	0.30^a^^b^	<0.001

For ethnic-specific popular channels, three popular channels were for Malay whilst five popular channels were Chinese, and four popular channels were Indian. Values in the same row sharing same superscript letters are significantly different. Dunn test: *p<*0.01.

a However, one TV channel was common to both Malay and Indian viewership.

### Persuasive techniques

**Fig. 3:**
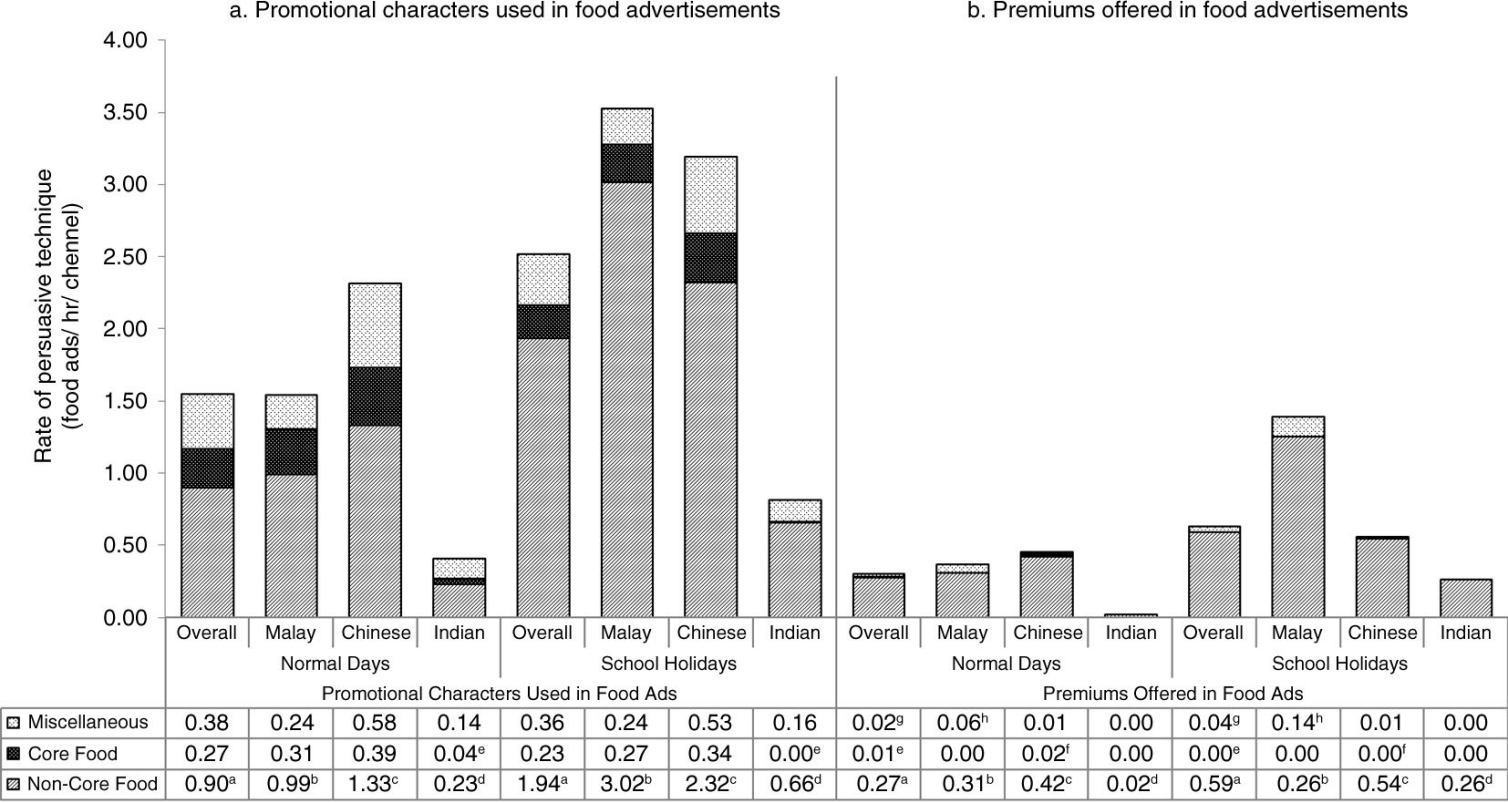
Persuasive techniques as per seasonal variation and TV channel ethnicity. (a) Promotional characters used in food advertisements. (b) Premiums offered in food advertisements. For ethnic-specific popular channels, three popular channels were for Malay whilst five popular channels were Chinese and four popular channels were Indian. One TV channel was common to both Malay and Indian viewership. Hence, overall analysis was carried out based on only 11 channels. Within each figure, values sharing same superscripts are significantly different between normal days and school holidays.

The most common persuasive technique used in TV food advertising was the use of promotional characters, and this trend was common to all ethnics channels selected. As indicated in Fig. 3, the rate of promotional characters used in food advertising was more relevant to non-core foods than core foods or miscellaneous food products. In contrast, the use of premium offer in food advertising was consistently low across both time periods. However, premium offers were more often associated with non-core foods.

## Discussion

This study identified higher rates of unhealthy food advertising occurred during school holiday periods compared to normal days for children. This is a marketing strategy because children would naturally have more free time to watch TV as suggested by Boyland et al. (31). Food advertising was pervasive throughout school holidays across all ethnic channels and mainly promoted sugar-sweetened drinks. A systematic review and meta-analysis has established a link between sugar-sweetened beverage consumption and increased risk of childhood obesity (32). A study in the United States indicated that every incremental increase of 100 advertisements of sugar-sweetened drinks was associated with a 9.4% increase in children's consumption of soft drinks (33). Thus, our finding on the high rate of sugary drink advertising is a serious concern in Malaysia if this is going to reflect an increased consumption of soft drinks by children.

Consistent with a previous study conducted in Malaysia in 2006 (21), the current findings indicated non-core foods were the most advertised food products. However, we detected a change in the type of food products most frequently advertised. Based on content analysis of TV food advertising data, Karupaiah et al. (21) identified unhealthy snacks as the most dominant advertised food products in contrast to the sugar-sweetened drinks reported in our study. This increment in advertising of sugar-sweetened drinks is also reported in Spain, India, and other Asia Pacific countries with this product being the most frequently advertised beverage on TV (29, 34, 35). From our observation of TV media, the increment in advertising for sugary drinks since 2006 (21) mostly originates from multinational companies, such as Coca-cola, Nestle, and Pepsico, as opposed to local brands.

Current Malaysian government guidelines restricting advertising and sponsorship by fast-food companies during children's TV programs apply during children's program targeting 4–9 year olds and when children's TV viewership exceeds 4% (22). Despite these restrictions, fast foods were one of the three most popular advertised non-core foods. We also noted that during children's PVT, the rate of fast-food advertisements was higher compared to NPVT (0.41 *vs* 0.24 food ads/hr/channel). This indicates current government regulations are limited in their ability to protect children from fast-food advertising on TV. The impact of this guideline is also limited by the lack of provisions related to other unhealthy food and beverages, particularly sugar-sweetened drinks. As demonstrated by viewership audience data in our study, the large majority of children watch TV outside of the targeted children's programs for which guidelines apply, with PVT occurring during the evening periods.

An alternative approach to monitoring and evaluating food advertising is by using children audience composition data as a standardised method to capture significant proportions of children exposed to marketing campaigns (1). A recent study suggested PVT reflected actual exposure of children to non-core food advertising (23). Based on our findings, the exposure of non-core foods was consistently higher during PVT compared to NPVT for children. The IFBA is now committed only to advertising products that meet specific nutritional criteria based on accepted national and international evidence and/or applicable national and international dietary guidelines in 2008 (20). However, our findings contradict a report from this alliance which found TV food and beverages advertising compliance was 99.2% (20). A reason for this discrepancy is that the IFBA code of practice only applies during TV viewing times when children make up at least 50% of the audience, which does not often occur in the real-world scenario (7). Further, the IFBA code is more permissive of foods that are actually inappropriate to be advertised to children, and is therefore relaxed about advertising of these foods. For instance, cereals containing sugar up to 35 g/100 g are deemed acceptable to be marketed to children, whereas expert opinion for healthy eating is that permitted foods should not exceed 20 g sugar/100 g (7).

We noted distinct differences in food advertising rates on Indian channels relative to Malay and Chinese channels. Most of the popular channels for Indian children were filtered satellite channels originating from India. Such foreign-origin advertisements are modified/removed as these are not relevant to the local market in Malaysia. It is also possible that these channels are less targeted by food companies, given the relatively low population numbers of this ethnic group in Malaysia compared to other ethnic groups (Malays or Chinese) as well as their lower household expenditures for these products (36). Conversely, non-core food advertising was found to be prevalent in Malay and Chinese channels and remarkably higher in Malay channels during school holidays. The non-core food advertising exposure pattern does not align with the reported patterns of childhood obesity by ethnicity in Malaysia (Chinese 26.6%>Indian 26.1%>Malay 18.9%) (19). However, obesity is well known to be a complex issue with multiple determinants (37). By using a mathematical simulation model, it was projected that TV food advertising contributed to 15–40% of obesity prevalence in the United States, and an absence of unhealthy food advertising on TV therefore could yield a reverse shift of proportion from overweight children to normal weight (38).

Promotional characters were commonly used as persuasive marketing technique for non-core foods in Malaysia, and this marketing practice was prevalent during school holidays compared to normal days. This is consistent with previous research which highlight that persuasive marketing techniques were mainly used in non-core food relative to core food advertisements (6, 39). On further examination of the non-core food category, we found sugar-sweetened drinks, breakfast cereals, extruded snacks, ice cream, and instant noodles were more likely to be using the persuasive techniques (data not shown). Promotional characters included branded cartoons or celebrities or famous actors who were company spokespersons for the non-core food products. The advertising impact of this technique is well documented to show associations with brand recognition, positive attitudes towards food products, and even brand loyalty at an earlier age (6, 40). Further, enhanced consumption of high-carbohydrate and high-fat foods by overweight and obese children is associated with the use of promotional characters during food advertising (41). Repetition of promotional characters from food advertising could transfer the positive effects related to characters (42) and even more, credibility of celebrities in their own field would be mistaken and further extended to the product they are endorsing (43).

Our results are constrained by the fact that this is a cross-sectional study. However, the selection of TV channels is valid to represent TV exposure patterns generally as the data includes normal days, weekdays, and weekend days, and at the same time provides data for all ethnic-specific popular TV channels. A major strength of this study is to provide a wider scope of understanding content analysis of food advertising on Malaysian TV channels by including seasonal variation as a factor and an improved model for classifying food codes previously standardised by researchers in a multicentre international study (35). It is important to note that in this study, the selection of TV channels was specific to ethnicity and ranked by children's preference as determined by Nielsen's TAM data. Further, persuasive techniques of marketing used in TV food advertising were assessed for the first time in Malaysia.

Based on our findings it is apparent that stringent regulation of TV food advertising is critical during PVT, which relates to children's viewership. The prior implementation of a content code that bans alcohol advertising in Muslim-majority Malaysia, which was successfully reflected in a zero rate detection of alcohol advertisements on TV, indicates that advertising content restrictions are possible. In contrast, our results also showed that current regulations in Malaysia are not able to protect children entirely from high rates of non-core food advertising on TV. Lastly, as highlighted by public health professionals, a standardised set of definitions for classifying TV food products according to health values, and specifying children's peak viewing period will enable children to be better protected, either directly or indirectly from exploitation by TV food marketing.

## Conclusions

This study revealed children's high exposure to non-core food advertising on Malaysian TV channels. Non-core food advertisements were shown four times more frequently during normal days than core foods, and the non-core food advertising rate doubled during school holidays. Food advertising exposure varied among channels popular with ethnic groups, suggesting policy to regulate advertising should factor ethnicity in the future. The high rate of advertising for sugary drinks warrants a further regulatory action by government to limit these advertisements.
